# Epidermal growth factor receptor promotes cerebral and retinal invasion by *Toxoplasma gondii*

**DOI:** 10.1038/s41598-018-36724-2

**Published:** 2019-01-24

**Authors:** Yalitza Lopez Corcino, Jose-Andres C. Portillo, Carlos S. Subauste

**Affiliations:** 1 Department of Pathology, Case Western Reserve University School of Medicine, Cleveland, Ohio USA; 2 Division of Infectious Diseases and HIV Medicine, Department of Medicine, Case Western Reserve University School of Medicine, Cleveland, Ohio USA

## Abstract

Little is known about strategies used by pathogens to facilitate CNS invasion. *Toxoplasma gondii* reaches the CNS by circulating in blood within leukocytes or as extracellular tachyzoites. *T. gondii* induces EGFR signaling *in vitro* during invasion of mammalian cells. We examined the effects of endothelial cell EGFR on CNS invasion. Transgenic mice whose endothelial cells expressed a dominant negative (DN) EGFR (inhibits EGFR signaling) exhibited diminished parasite load and histopathology in the brain and retina after *T. gondii* infection. I.V. administration of infected leukocytes or extracellular tachyzoites led to reduced parasite loads in mice with DN EGFR. This was not explained by enhanced immunity or reduced leukocyte recruitment. Endothelial cell infection is key for CNS invasion. Parasite foci in brain endothelial cells were reduced by DN EGFR. DN EGFR in these cells led to recruitment of the autophagy protein LC3 around *T. gondii* and spontaneous parasite killing dependent on the autophagy protein ULK1 and lysosomal enzymes. The autophagy inhibitor 3-MA prevented DN EGFR mice from exhibiting reduced CNS invasion. Altogether, EGFR is a novel regulator of *T. gondii* invasion of neural tissue, enhancing invasion likely by promoting survival of the parasite within endothelial cells.

## Introduction

*Toxoplasma gondii* is an obligate intracellular protozoan of worldwide distribution that infects humans and warm-blooded animals^1^. *T. gondii* causes a chronic infection even in immunocompetent hosts that is characterized by the presence of tissue cysts. Approximately 30% of the world population is chronically infected with *T. gondii*, making this pathogen the most common cause of a chronic parasitic infection in the world^1^. The retina and brain are the main tissues affected in toxoplasmosis^1^. While cerebral toxoplasmosis occurs in immunosuppressed hosts, ocular toxoplasmosis can also occur in immunocompetent individuals^1^. Moreover, ocular toxoplasmosis is a major cause of infectious retinitis in the world^2^.

*T. gondii* invades the brain and retina through a hematogenous route^3–5^. The parasite circulates in blood within infected leukocytes and also as extracellular tachyzoites^3–5^. Three mechanisms for parasite invasion through the blood-brain and blood-retinal barrier have been proposed^6^: (i) Paracellular entry whereby extracellular tachyzoites transmigrate through tight junctions between endothelial cells; (ii) Transmigration of infected leukocytes across the endothelial cell layer (“Trojan horse” mechanism); (iii) Transcellular entry whereby endothelial cells become infected, enabling release of *T. gondii* in the neural parenchyma. Recent studies indicate that the last mechanism appears to be the most important^5^. Neural endothelial cells become infected during parasite dissemination^5^. Replication of *T. gondii* within these cells leads to parasite egress into the brain parenchyma^5^. Indeed, infected endothelial cells are considered a central portal of parasite entry into neural tissue^5^. While the mechanisms of parasite invasion into the CNS have been studied, little is known about factors that regulate parasite invasion of neural tissues.

Recent studies revealed that *T. gondii* activates host cell Epidermal Growth Factor Receptor (EGFR) during the process of invasion^7,8^. EGFR activation allows *T. gondii* to survive within host cells by avoiding autophagy-dependent lysosomal degradation of the parasite^7,8^. We examined whether EGFR modulates *T. gondii* invasion of the brain and retina using transgenic mice that express in endothelial cells a dominant negative (DN) mutant of EGFR that lacks the intracytoplasmic domains and inhibits EGFR signaling. Blockade of EGFR led to spontaneous killing of *T. gondii* within endothelial cells, reduction in the foci of infected endothelial cells *in vivo*, decreased parasite load in the brain and retina as well as protection against cerebral and ocular toxoplasmosis. The work herein uncovered that EGFR plays a critical role in facilitating parasite invasion of the brain and retina.

## Results

### Transgenic mice that express DN EGFR in endothelial cells

We used a binary tetracycline (Tet) repressible system to generate mice that express DN EGFR in endothelial cells. The driver line were heterozygous mice expressing the tetracycline (Tet)-repressible transactivator (tTA) under the control of the widely used endothelial promoter *Tie1* (Tie1-tTA mice)^9,10^ (Supplementary Fig. 1a). The responder line consisted of homozygous mice containing DN mutant of EGFR cloned downstream of a tetracycline operator (*tet°*; Tet°-DN EGFR)^11^. DN EGFR was a truncated form of the receptor that lacks the entire protein tyrosine kinase and carboxyl-terminal domains^11^. This truncated EGFR mutant forms heterodimers with WT EGFR, inhibiting EGF-dependent signaling^12^. After mating Tie1-tTA mice with Tet° DN-EGFR animals, double transgenic offspring are predicted to express DN EGFR in endothelial cells, while no expression is expected in single transgenic mice (carrying Tet^O^ DN-EGFR only). Mice were given doxycycline during breeding in order to turn off expression of DN EGFR. Doxycycline was removed after birth to enable expression of DN EGFR. PCR analysis of genomic DNA of the offspring identified single transgenic (Trg-Ctr) or double transgenic (Trg-DN EGFR) mice (Supplementary Fig. 1b). Lung CD31^+^ and CD31^−^ cells were isolated from Trg-Ctr and Trg-DN EGFR mice. All cell preparations expressed WT EGFR (MW 175 kD, detected by an anti-EGFR Ab directed against intracytoplasmic tail of EGFR) (Supplementary Fig. 1c). In contrast, only CD31^+^ cells (endothelial cells) from Trg-DN EGFR mice expressed the truncated form of EGFR (MW 115 kD, detected by an anti-EGFR Ab directed against extracellular domain of EGFR) (Supplementary Fig. 1c). CD31^−^ cells from Trg-Ctr and Trg-DN EGFR mice lacked truncated EGFR. Truncated EGFR was expressed in brain endothelial cells from Trg-DN EGFR mice while this molecule was not detected in lysates from brain endothelial cells from Trg-Ctr mice (Supplementary Fig. 1d). Moreover, as reported for primary bone marrow cells^13^, splenocytes lacked detectable expression of EGFR (Supplementary Fig. 1d). Next, we examined EGFR signaling. Brain endothelial cells from Trg-Ctr and Trg-DN EGFR mice were incubated with EGF. Immunoblots were performed using Abs that detect only WT EGFR: anti-EGFR Ab directed against intracytoplasmic tail of EGFR and anti-phospho Y1068 EGFR (truncated EGFR lacks intracytoplasmic domains). EGFR signaling (Y1068 autophosphorylation) was inhibited in endothelial cells from Trg-DN EGFR (Supplementary Fig. 1e). Altogether, Trg-DN EGFR mice expressed the truncated form of EGFR in endothelial cells leading to inhibition of EGFR signaling.

### Expression of DN EGFR reduces parasite load and histopathology in the eye and brain

**Figure 1:**
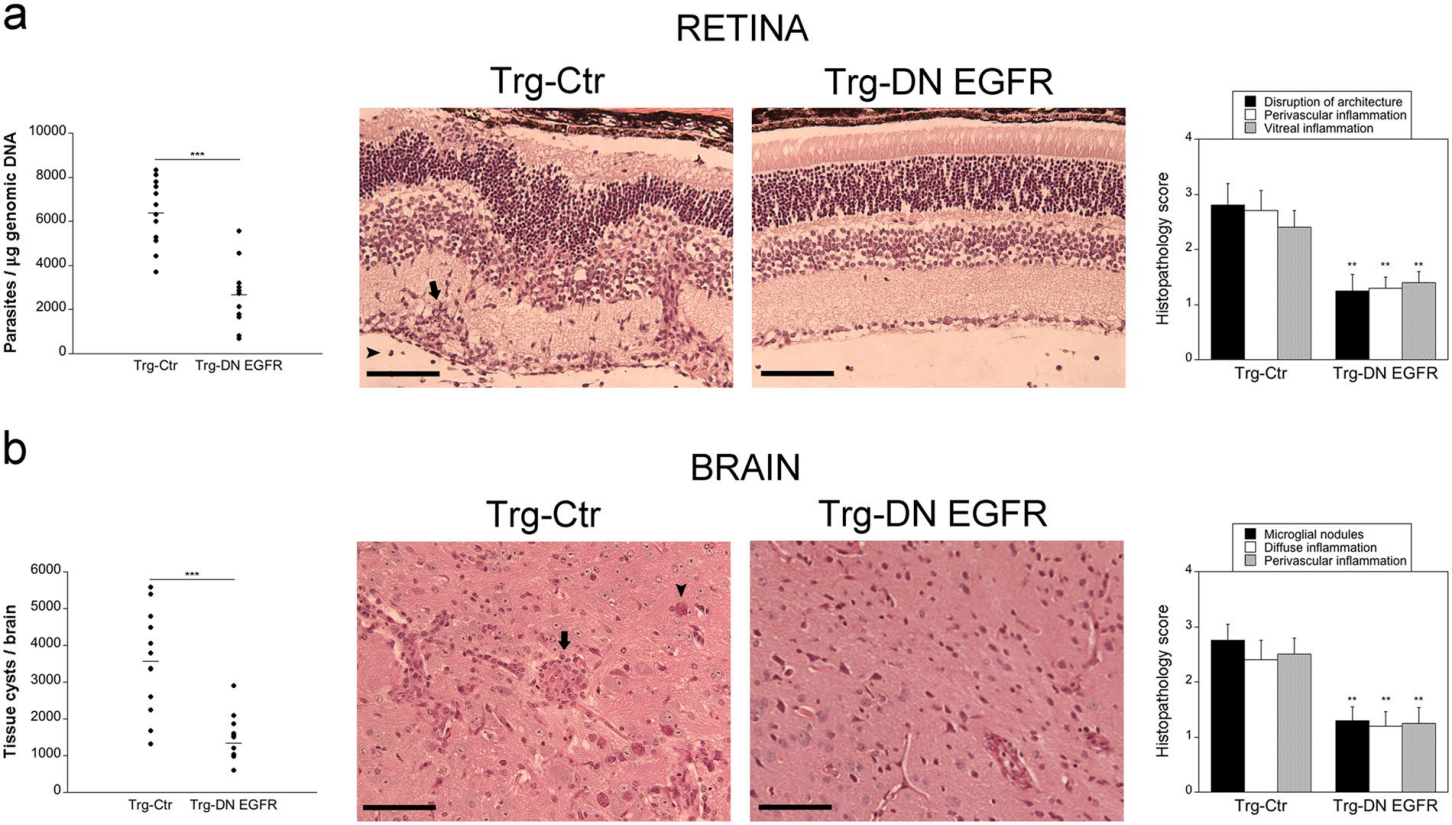
Expression of DN EGFR diminishes *T. gondii* load in the eye and brain and enhances resistance to ocular and cerebral toxoplasmosis. Trg-Ctr and Trg-DN EGFR mice were infected with tissue cysts of the ME49 strain of *T. gondii* and euthanized at 14 days. (**a**) Retinal levels of *T. gondii B1* gene were examined using qPCR. 12 mice per group pooled from 3 independent experiments. Eyes from infected Trg-Ctr showed disruption of retinal architecture, perivascular (arrow) and vitreal inflammation (arrowhead). H&E; X200. Bar, 100 μm. Histopathologic changes in the retina and brain were scored using previously described criteria (refs ^33,34^). Bars are mean ± SEM of 12 mice per group from 3 pooled experiments. (**b**) *T. gondii* tissue cysts per brain. 12 mice per group pooled from 3 independent experiments. Brains from Trg-Ctr mice showed more prominent microglial nodules (arrow) and frequent tissue cysts (arrowhead). PASH original magnification x200. Bar, 100 μm. **p < 0.01, ***p < 0.001 (Student’s *t* test; Mann-Whitney *U* test).

Studies of parasite dissemination after infection of type II strains of *T. gondii* revealed that both i.p. or oral routes of infection lead to rapid parasite seeding of the spleen, liver and lung^14,15^, that is followed by invasion of the brain and retina^3,14–16^. The timing for hematogenous seeding of neural tissue is similar in both instances^16^. Thus, both routes of infection are suitable to study how hematogenous invasion of the eye and brain is regulated. WT, Trg-Ctr and Trg-DN EGFR mice were infected with 30 ME49 *T. gondii* tissue cysts i.p. While parasite load in the spleen, lung and liver were similar in all groups of mice (Table 1), Trg-DN EGFR mice exhibited lower parasite load in the eye and brain (Table 1 and Fig. 1). This was accompanied by decreased disruption of the retinal architecture, reduction in perivascular and vitreal inflammation, as well as decreased brain parenchymal inflammatory foci and perivascular cuffing in Trg-DN EGFR mice (Fig. 1). Taken together, expression of DN EGFR enhanced protection against ocular and cerebral toxoplasmosis.

**Table 1:** *T. gondii* parasite load in WT, Trg-Ctr (Ctr) and Trg-DN EGFR (DN) mice.

	Day 3			Day 7			Day 14		
	WT	Ctr	DN	WT	Ctr	DN	WT	Ctr	DN
Spleen	241 ± 35	218 ± 56	213 ± 51	10,042 ± 1,880	9,567 ± 2,366	9,127 ± 2,557	320 ± 41	409 ± 51	353 ± 37
Liver	105 ± 25	80 ± 18	72 ± 16	3,746 ± 410	4,062 ± 453	3,768 ± 318	130 ± 25	191 ± 60	142 ± 90
Lung	28 ± 5	24 ± 4	18 ± 7	3,625 ± 710	3,416 ± 416	3,189 ± 385	1,425 ± 175	1,270 ± 81	968 ± 157
Brain	Undetec	Undetec.	Undetec	1,170 ± 150	1,010 ± 139	371 ± 40**	33,250 ± 1,900	38,380 ± 2,258	15,352 ± 698***
Eye	Undetec	Undetec	Undetec	251 ± 32	244 ± 35	88 ± 14**	6,560 ± 510	6,397 ± 437	2,674 ± 406***

Mice were infected i.p. with tissue cysts of the ME49 strain of *T. gondii*. Genomic DNA was isolated and levels of the *B1* gene of *T. gondii* were examined by quantitative PCR. A standard curve of DNA from known numbers of parasites per reaction was used to calculate the number of parasites per μg of genomic DNA isolated from organs. Results are shown as the mean ± SEM of 6–12 mice pooled from 2–3 independent experiments. **p < 0.01; ***p < 0.001 (Student’s *t* test).

### Expression of DN EGFR does not affect cellular and humoral immunity against *T. gondii*

Serum levels of IL-12 p40, IFN-γ and TNF-α (Supplementary Fig. 2a) in addition to *in vitro* secretion of these cytokines were similar between Trg-Ctr and Trg-DN EGFR mice (Supplementary Fig. 2b). Similarly, the expression of nitric oxide and Irgm3, mediators of protection induced by IFN-γ, were similar in Trg-Ctr and Trg-DN EGFR mice (Supplementary Fig. 2c,d). Expression of DN EGFR did not affect the induction of *T. gondii*-reactive IFN-γ-producing CD4^+^ and CD8^+^ T cells (Supplementary Fig. 2e). Moreover, Trg-Ctr and Trg-DN EGFR mice had similar serum anti-*T. gondii* IgG titers (log_2_ antibody titers: 10.44 ± 0.2 vs. 11 ± 0.25; n = 5; p > 0.05).

**Figure 2:**
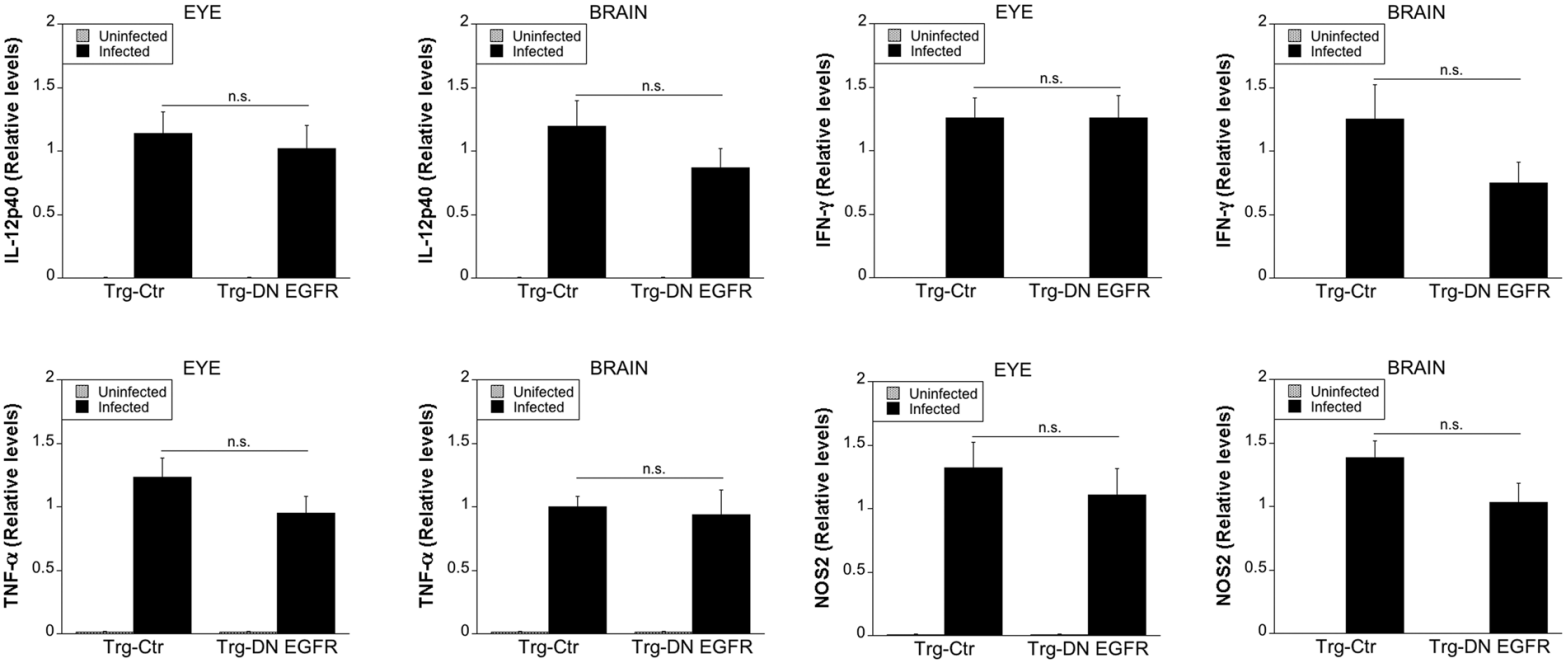
Effect of DN EGFR on the expression of IL-12, IFN-γ, TNF-α and NOS2 in the eye and brain. Trg-Ctr and Trg-DN EGFR mice infected with ME49 tissue cysts were euthanized at 14 days. Uninfected mice were used as controls. Levels of IL-12 p40, IFN-γ, TNF-α and NOS2 mRNA in eyes and brains were examined using qPCR. Levels were compared to those of one Trg-Ctr mouse that was given an arbitrary value of 1. Each group contained 12 mice pooled from 3 experiments. Results are shown as the mean ± SEM.

We examined expression of IL-12 p40, IFN-γ, TNF-α and NOS2 in the eye and brain. mRNA levels of these molecules were not higher in infected Trg-DN EGFR mice compared to infected Trg-Ctr mice (Fig. 2). Altogether, enhanced resistance against ocular and cerebral toxoplasmosis in Trg-DN EGFR mice occurred without an increase in local or systemic expression of immune mediators of resistance.

### Expression of DN EGFR restricts invasion of the retina and brain by *T. gondii*

**Figure 3:**
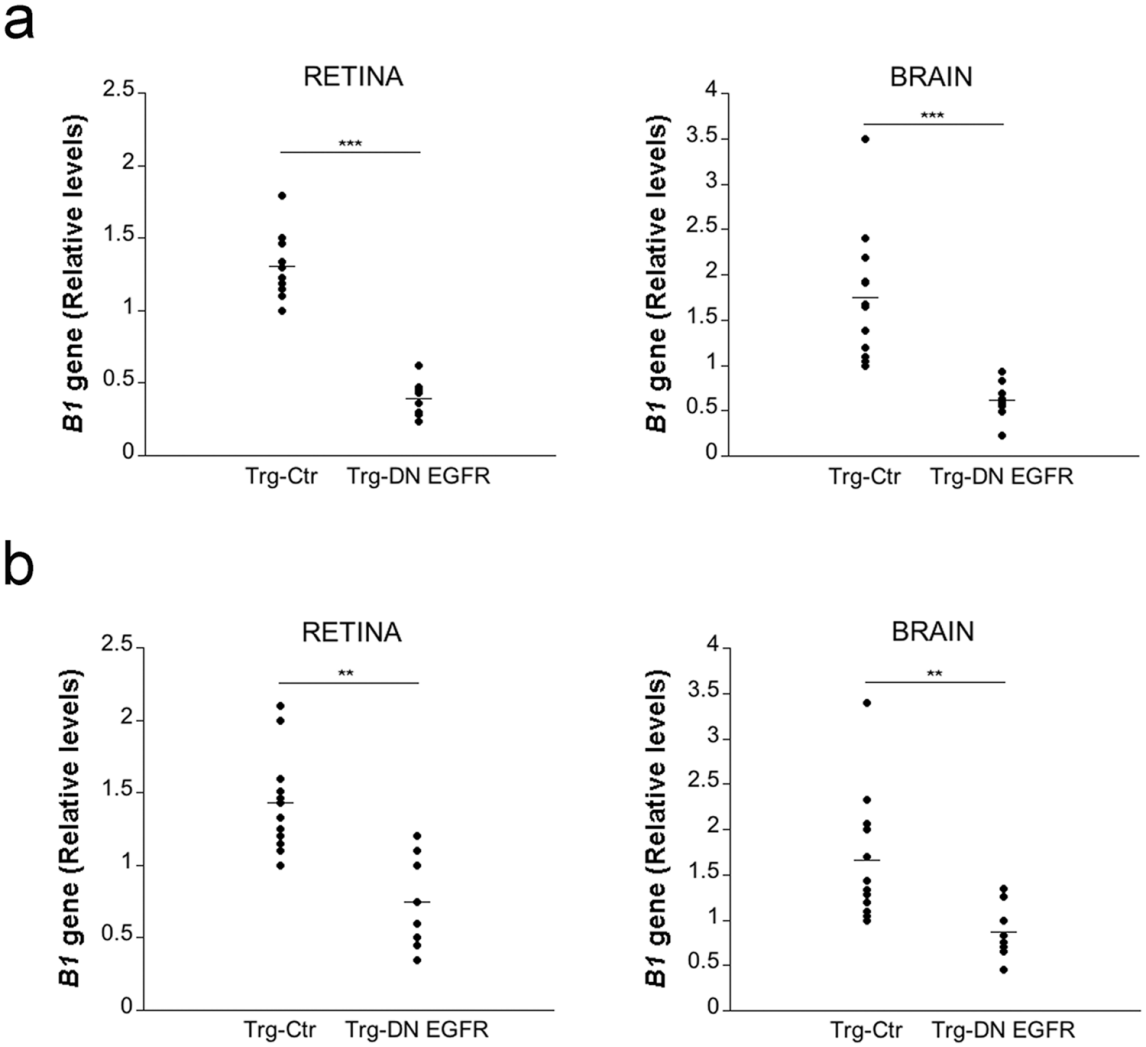
Expression of DN EGFR diminishes hematogenous invasion of the eye and brain by *T. gondii*. (**a**) Dendritic cells infected *in vitro* with PTG *T. gondii* were injected i.v. to Trg-Ctr or Trg-DN EGFR mice. Retinas and brains were obtained 1 d after i.v. challenge. Expression of *B1* gene was assessed by qPCR. Levels were compared to those of one Trg-Ctr mouse that was given an arbitrary value of 1. (**b**) Mice were injected i.v. with 5 × 10^5^ PTG tachyzoites. *T. gondii B1* gene was examined 1 day post-challenge and assessed as above. 8–12 mice per group pooled from 2–3 independent experiments. **p < 0.01; ***p < 0.001 (Student’s *t* test).

*T. gondii* reaches the brain and retina hematogenously, and the parasite circulates in the blood within infected leukocytes (including monocytes and dendritic cells)^3–5^ or as extracellular tachyzoites^5^. Parasite load in the blood was similar in Trg-Ctr and Trg-DN EGFR mice (Relative levels of *T. gondii B1* gene on day 5: Trg-Ctr 1.18 ± 0.3; Trg-DN-EGFR 1.21 ± 0.2; n = 5). Thus, we used models of i.v. challenge to directly examine whether expression of DN EGFR impairs parasite invasion of the eye and brain. First, we used infected dendritic cells as a model of cells that carry intracellular tachyzoites since dendritic cells traffic into the brain of *T*. gondii-infected mice^17^ and adoptive transfer of infected dendritic cells leads to rapid dissemination of infection into the CNS^4^. Mouse dendritic cells infected *in vitro* with tachyzoites of PTG *T. gondii* were injected i.v. into mice. Compared to Trg-Ctr mice, Trg-DN EGFR mice exhibited markedly lower parasite load in the retina and brain (Fig. 3a). Although reported to be less efficient than circulating infected leukocytes^4,18^, extracellular tachyzoites present in the blood can invade the brain^5^. Trg-DN EGFR mice also had lower parasite loads in the retina and brain after i.v. administration of PTG *T. gondii* tachyzoites (Fig. 3b). Taken together, the presence of DN EGFR diminished invasion of the retina and brain by *T. gondii*.

### Leukocyte migration into neural tissue is not altered in Trg-DN EGFR mice

**Figure 4:**
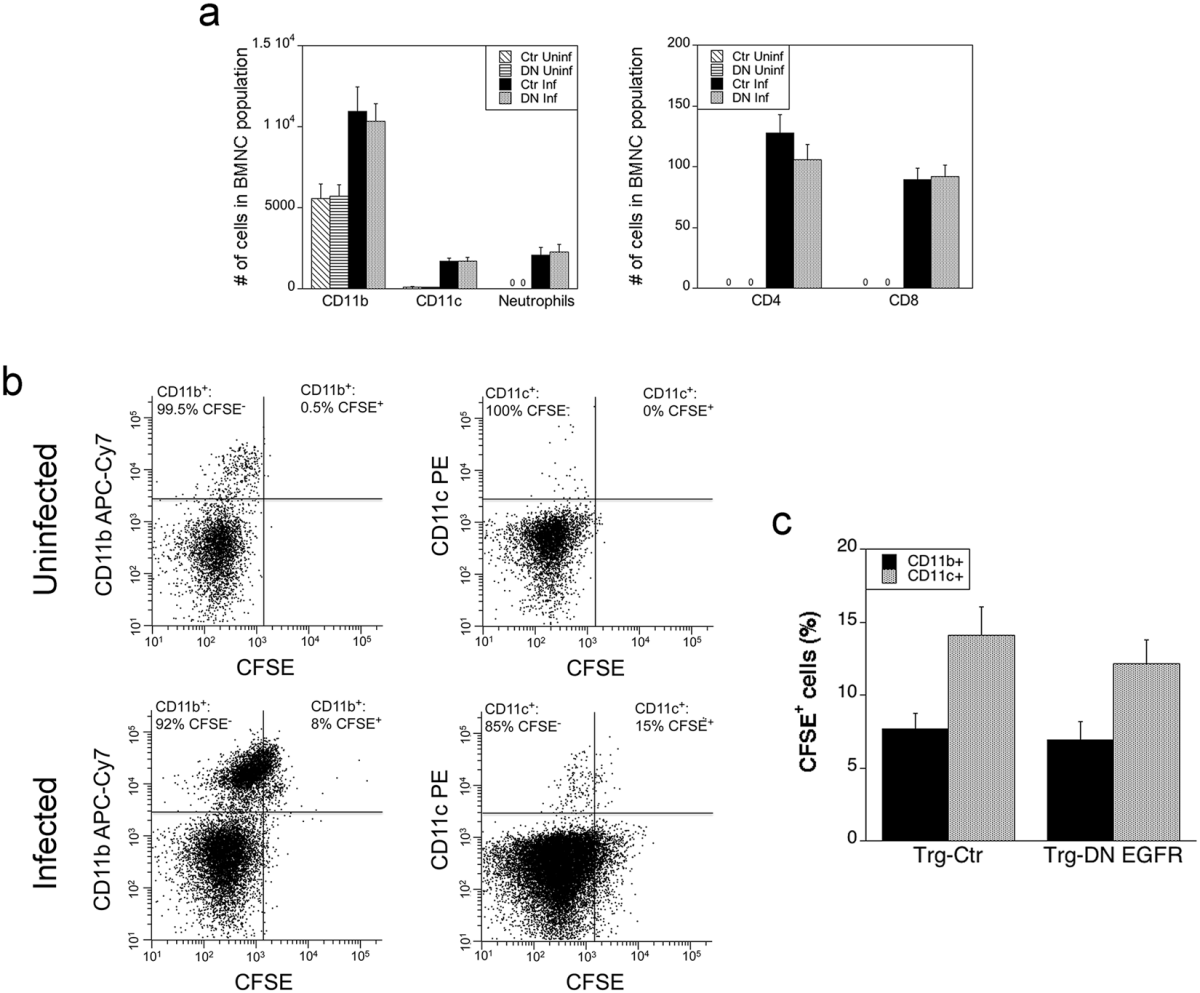
Expression of DN EGFR does not affect recruitment of leukocytes or impair transmigration of leukocytes into the brain. (**a**) Mice were infected with 30 ME49 tissue cysts and euthanized 7 days after infection. Uninfected mice were used as controls. **(a**) The numbers of BMNC that were CD11b^+^, CD11c^+^, CD45^+^ F4/80^−^ Gr-1^+^ (neutrophils), CD3^+^ CD4^+^ or CD3^+^ CD8^+^ were determined using flow cytometry. (**b**,**c**) Mice were injected with CFSE i.v. 5 days after infection with ME49 tissue cysts. Uninfected mice injected with CFSE were used as controls. Expression of CFSE on CD11b^+^ and CD11c^+^ cells was examined in BMNC isolated 2 days after CFSE injection. (**b**) Gate to identify cells that stained with CFSE was obtained using BMNC of mice that were not injected with CFSE. Dot plots represent data obtained from representative uninfected and infected Trg-Ctr mice. Numbers shown in dot plots represent the percentages of either CD11b^+^ cells or CD11c^+^ cells that were CFSE^+^ or CFSE^−^. (**c**) Bar graph shows mean ± SEM of percentages of CD11b^+^ and CD11c^+^ cells that were CFSE^+^ in infected Trg-Ctr and Trg-DN EGFR mice. Total numbers of BMNC per mouse and the percentages of CD11b^+^ or CD11c^+^ cells were similar between infected Trg-Ctr and infected Trg-DN EGFR mice. Bars are mean ± SEM of 9 samples per group pooled from 2 independent experiments.

Monocytes, dendritic cells, neutrophils and T cells in peripheral blood contain intracellular *T. gondii*, and monocytes as well as dendritic cells are reported to facilitate parasite invasion of the CNS^1–3^. We analyzed the phenotypic composition of brain mononuclear cells (BMNC) to determine if enhanced protection in Trg-DN EGFR mice may be due to changes in leukocyte recruitment. Analysis was conducted on day 7 post-infection, a time point when there is already decreased *T. gondii* load in the brain of Trg-DN EGFR mice. The recruitment of CD11b^+^, CD11c^+^, Gr-1^+^ F4/80^−^ (neutrophils), CD3^+^ CD4^+^ and CD3^+^ CD8^+^ cells were similar in Trg-Ctr and Trg-DN EGFR mice (Fig. 4a). Thus, the reduction in parasite load in the brain of DN-EGFR mice occurred without changes in recruitment of leukocytes.

To further assess whether expression of DN EGFR impaired transmigration of circulating leukocytes into the brain, we injected mice with CFSE i.v. This approach labels peripheral blood leukocytes *in vivo* but does not directly label brain parenchymal cells in *T. gondii*-infected mice and has been shown to detect subsequent transmigration of CFSE-labeled leukocytes into the brain of these mice^3^. Trg-Ctr and Trg-DN EGFR mice received CFSE i.v. 5 days after infection with *T. gondii*^3^. As previously reported^3^, CD11b^+^ and CD11c^+^ cells that were CFSE^+^ were detected in BMNC from *T. gondii*-infected mice when BMNC were collected 2 days after CFSE injection (Fig. 4b). Infected Trg-Ctr and Trg-DN EGFR mice had similar percentages of CFSE^+^ cells among the CD11b^+^ and CD11c^+^ populations in BMNC (Fig. 4c). Together, these studies indicate that it is unlikely that restriction of transmigration of infected leukocytes explained the protective effect of DN EGFR in neural tissue.

### Parasite foci in neural endothelial cells are reduced in Trg-DN EGFR mice

**Figure 5:**
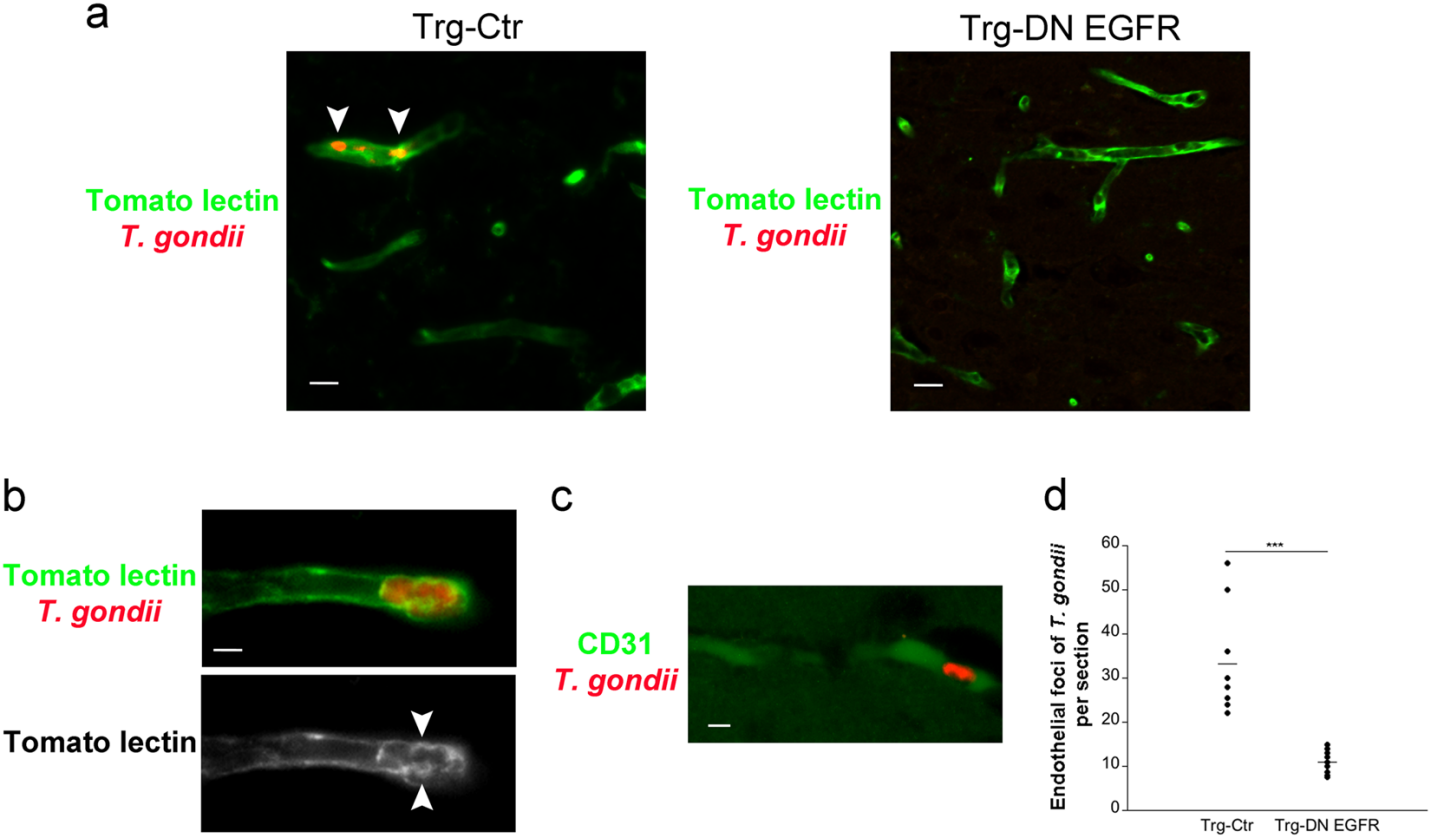
Expression of DN EGFR diminishes *T. gondii* foci within brain endothelial cells *in vivo*. Mice received *T. gondii*-infected dendritic cells i.v. After 4 d mice were perfused. (**a**,**b**) Brain sections were stained with anti-*T. gondii* Ab and Tomato lectin (efficiently labels neural endothelial cells). Clusters of *T. gondii* parasites within tomato lectin^+^ elongated structures (endothelial cells) were examined. Endothelial cells contained foci of parasites (arrowheads). (**b**) A parasite focus that appeared located within vacuoles (arrowheads). Images shown were obtained from representative Trg-Ctr mice (X400). Bar, 10 μm (**a**), 5 μm (**b**). (**c**) Brain section from a representative Trg-Ctr mice stained with anti-*T. gondii* and anti-CD31 Abs (X400). Bar, 10 μm (**d**) Numbers of endothelial foci of *T. gondii* per coronal section. Bars are mean ± SEM of 7–9 mice per group from 2 pooled experiments. ***p < 0.001 (Student’s *t* test).

Infection of endothelial cells followed by parasite egress into neural parenchyma are key for parasite entry into the CNS^5^. We determined whether the presence of DN EGFR in endothelial cells reduces focal areas of parasites in endothelial cells. Trg-Ctr and Trg-DN EGFR underwent i.v. injection of *T. gondii*-infected dendritic cells. Focal areas of parasites were noted in brain endothelial cells identified by staining with Tomato lectin or anti-CD31 mAb (Fig. 5a–c). Parasites appeared to be located within vacuoles in these cells (Fig. 5b). The numbers of parasite foci within endothelial cells were significantly reduced in Trg-DN EGFR mice (Fig. 5d). Thus, expression of DN EGFR in endothelial cells reduced the foci of infected endothelial cells.

### Brain endothelial cells from Trg-DN EGFR mice spontaneously kill the parasite through autophagy and this process appears to contribute to decreased parasite invasion of the CNS

**Figure 6:**
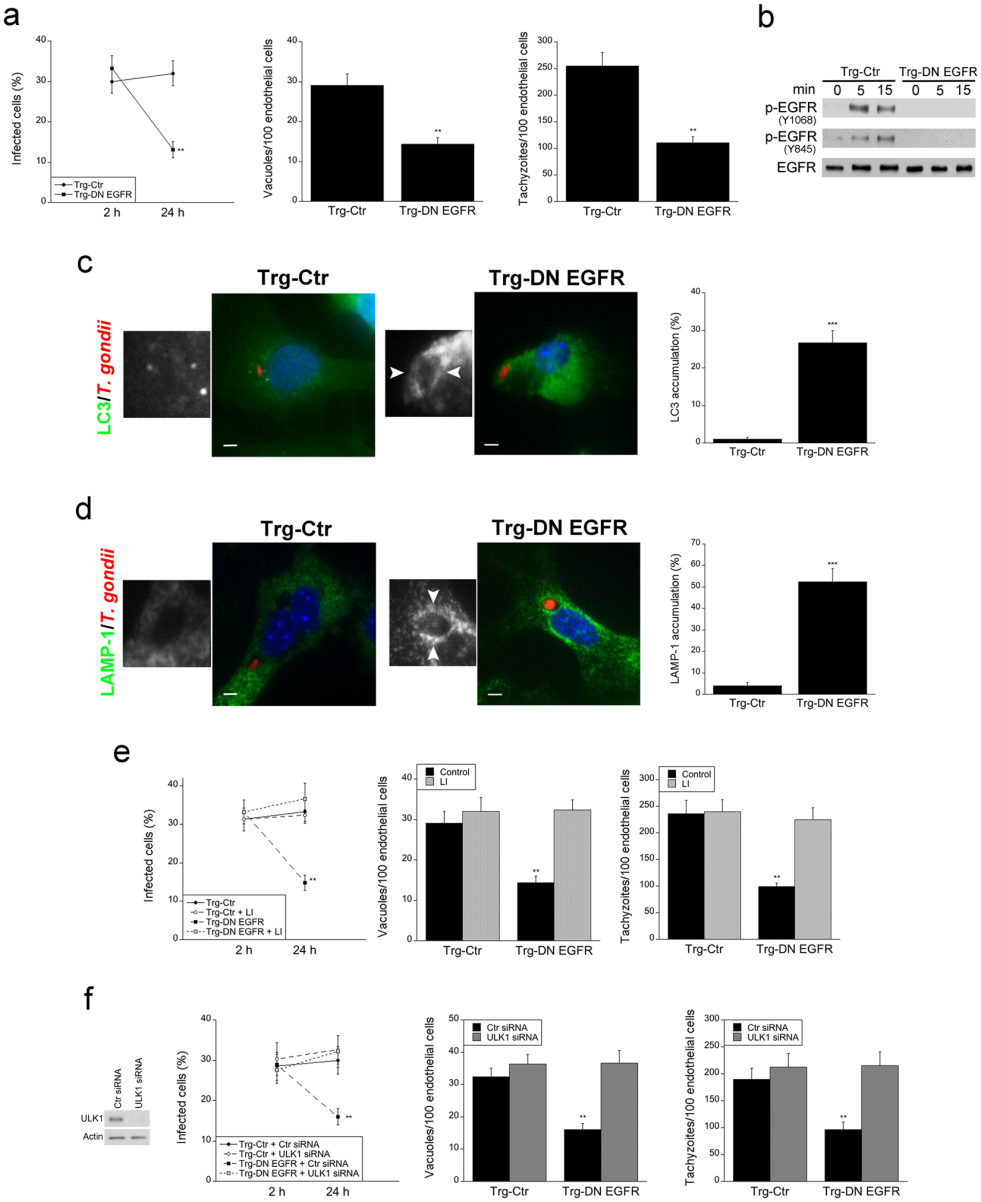
Brain endothelial cell from Trg-DN EGFR exhibit spontaneous targeting of intracellular *T. gondii* by LC3 and LAMP-1 as well as killing of the parasite dependent on autophagy. (**a**) Brain endothelial cells from Trg-Ctr and Trg-DN EGFR mice were infected with RH *T. gondii*. Monolayers were assessed by light microscopy. The percentages of infected cells, numbers of vacuoles containing *T. gondii* per 100 endothelial cells and tachyzoites per 100 endothelial cells were determined at 2 and 24 h. (**b**) Brain endothelial cells from Trg-Ctr and Trg-DN EGFR mice were infected with RH *T. gondii*. Lysates obtained at 0, 5 and 15 min post-challenge were subjected to immunoblot as indicated. (**c**,**d**) Brain endothelial cells from Trg-Ctr and Trg-DN EGFR mice were infected with RFP *T. gondii* (RH). Expression of LC3 was assessed by immunofluorescence 5 h after challenge (**c**). Insets represent magnification of areas around the parasites. Arrowheads indicate LC3 accumulation around the parasite (X630). Bar, 5 μm. (**d**) Expression of LAMP-1 was assessed by immunofluorescence 8 h after challenge. Arrowheads indicate LAMP-1 accumulation around the parasite. Bar, 5 μm. Bar graphs represent percentages of parasitophorous vacuoles surrounded by ring-like accumulation of LC3 or LAMP-1. (**e**) Endothelial cells were challenged with RH *T. gondii* followed by addition of lysosomal inhibitors (LI; leupeptin and pepstatin). Cells were examined as above. (**f**) Endothelial cells were transfected with control or ULK1 siRNA followed by challenge with RH *T. gondii*. Bars are mean ± SEM of 6 samples per group pooled from 2–3 experiments. **p < 0.01; *** *p* < 0.001 (Student’s *t* test).

Given the *in vivo* reduction in foci of infection in endothelial cells from DN EGFR mice, we examined whether neural endothelial cells from these animals show spontaneous toxoplasmacidal activity. Primary brain endothelial cells from Trg-Ctr and Trg-DN EGFR mice were isolated and challenged with tachyzoites of *T. gondii*. Whereas the percentages of infected cells were similar in both groups of mice at 2 h post-challenge, endothelial cells from Trg-DN EGFR mice exhibited a marked reduction in the percentages of infection at 24 h (Fig. 6a). This was accompanied by a reduction in the numbers of parasite-containing vacuoles and the numbers of tachyzoites per 100 endothelial cells (Fig. 6a). Thus, expression of DN EGFR in neural endothelial cells resulted in spontaneous killing of *T. gondii*.

*T. gondii* activates EGFR in host cells to avoid being targeted by autophagy, a constitutive process of lysosomal degradation. Through parasite micronemal proteins (MIC) 3 and 6 (adhesins that contain Epidermal Growth Factor (EGF)-like domains, *T. gondii* causes EGFR autophosphorylation in mammalian cells^7^. In addition, during invasion of mammalian cells, *T. gondii* activates Focal Adhesion Kinase and its interacting partner Src, leading to transactivation of EGFR (Y845 phosphorylation)^8^. Both signaling cascades enable the parasite to avoid targeting by autophagosomes allowing its survival^7,8^. Thus, we examined *T. gondii*-induced EGFR autophosphorylation and Y845 EGFR phosphorylation in neural endothelial cells from Trg-Ctr and Trg-DN EGFR mice. Both Y1068 and Y845 EGFR phosphorylation were impaired in brain endothelial cells from Trg-DN EGFR mice infected with *T. gondii* (Fig. 6b). Next, we examined the expression of the autophagosome marker LC3 in *T. gondii*-infected brain endothelial cells. LC3 was recruited around the parasite in endothelial cells from Trg-DN EGFR mice whereas no such recruitment was noted in cells from Trg-Ctr animals (Fig. 6c). Autophagic killing of *T. gondii* is dependent on vacuole-lysosomal fusion and lysosomal enzymes. Indeed, LAMP-1 was recruited around the parasite in endothelial cells from Trg-DN EGFR mice (Fig. 6d) and killing of *T. gondii* observed in these cells was markedly impaired by incubation with lysosomal inhibitors (Fig. 6e). In addition, killing of *T. gondii* in Trg-DN EGFR endothelial cells was also abrogated by knockdown of Unc-51-like kinase 1 (ULK1) (Fig. 6f), an upstream inducer of canonical autophagy^19,20^.

**Figure 7:**
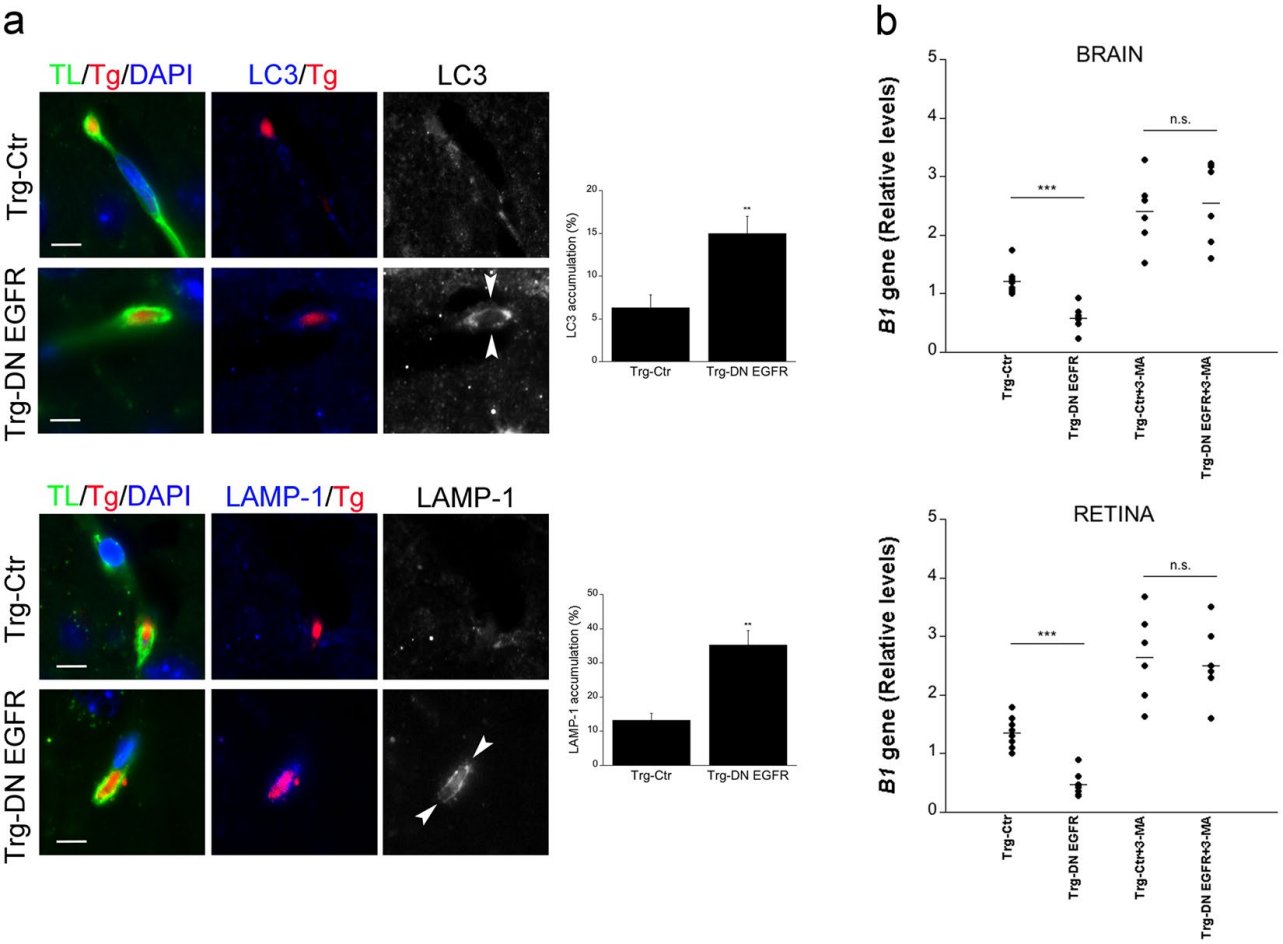
*In vivo* accumulation of LC3 and LAMP-1 around *T. gondii* in brain endothelial cells and effects of administration of an inhibitor of autophagy on hematogenous invasion of the brain and eye by *T. gondii*. (**a**) Mice received *T. gondii*-infected dendritic cells i.v. After 18 h, mice were perfused and brain sections were stained with Tomato lectin-DyLight 488, anti-*T. gondii* Ab plus Alexa 568-conjugated secondary Ab and anti-LC3 or anti-LAMP-1 Abs plus Alexa 647-conjugated secondary Abs. Images to the left show parasites present in Tomato lectin^+^ cells (endothelial cells). The images from the Trg-Ctr mouse show a parasite not surrounded by accumulation of LC3 or LAMP-1. Ring-like accumulation of LC3 or LAMP-1 around parasite (arrowheads) is noted in images from the Trg-DN EGFR mouse (X630). Bar, 5 μm. Bar graphs represent percentages of parasites present in endothelial cells that were surrounded by accumulation of LC3 or LAMP-1 (mean ± SEM from 2 pooled experiments). **p < 0.01 (Student’s *t* test). (**b**) Mice that received *T. gondii*-infected dendritic cells i.v were treated with the autophagy inhibitor 3-methyl adenine (3-MA) or vehicle as described in Methods. Brains and retinas were obtained 1 d after i.v. challenge. Expression of *B1* gene was assessed by qPCR. Levels were compared to those of one vehicle-treated Trg-Ctr mouse that was given an arbitrary value of 1. Results are pooled from 2 experiments. ***p < 0.001 (ANOVA).

Next, we examined the *in vivo* recruitment of LC3 and LAMP-1 around parasites in brain endothelial cells. Trg-Ctr and Trg-DN EGFR received *T. gondii*-infected dendritic cells i.v. A significant increase in the percentages of parasites surrounded by LC3 or LAMP-1 were noted in brain endothelial cells from Trg-DN EGFR (Fig. 7a). Finally, we explored the *in vivo* role of autophagy in the regulation of parasite invasion of the CNS. Trg-Ctr and Trg-DN EGFR mice that received infected dendritic cells i.v. were treated with or without 3-methyl adenine (3-MA), an inhibitor of autophagosome formation that prevents autophagic killing of *T. gondii*^7,21,22^. This was followed by assessment of parasite load at 24 h by PCR. *In vivo* treatment with 3-MA prevented Trg-DN EGFR from exhibiting reduced parasite load in the brain and retina (Fig. 7b). The effect of 3-MA was unlikely to be explained by increasing parasite load in peripheral tissues since *T. gondii* DNA was not detectable in the blood at the time of organ collection. Taken together, endothelial cells from Trg-DN EGFR mice spontaneously recruited LC3 around *T. gondii*, killing the parasite via autophagy *in vitro*. *In vivo* studies suggest that increased autophagic targeting occurs in endothelial cells from Trg-DN EGFR and support that the autophagy pathway may restrict parasite invasion of the CNS in Trg-DN EGFR mice.

## Discussion

While the mechanisms by which *T. gondii* invades the CNS have been identified, it is not known how host-pathogen interaction at the level of endothelial cells modulates *T. gondii* invasion of the CNS. Our studies uncovered EGFR expressed in endothelial cells as an important regulator of invasion of the brain and retina by *T. gondii*. Expression in endothelial cells of a DN EGFR that inhibits EGFR signaling led to a decrease in *T. gondii* invasion of the brain and retina as well as diminished development of encephalitis and retinitis. This protective effect was not mediated by enhanced cellular or humoral immunity against *T. gondii* or by a decrease in recruitment of leukocytes into neural tissue. In addition, DN EGFR did not impair the ability of *T. gondii* to infect endothelial cells. Rather, DN EGFR reduced parasite foci within endothelial cells *in vivo*, an effect that was lost by administration of an inhibitor of autophagy. Our studies revealed that *T. gondii* exploits EGFR in endothelial cells to promote invasion of the brain and retina, and suggest that this effect may be mediated by blocking the ability of endothelial cells to display of autophagic killing of the parasite.

EGFR is composed of extracellular (ligand binding), transmembrane, intracellular tyrosine kinase and carboxyl-terminal tail domains^23^. Ligand binding to the extracellular domain causes a conformational change in the kinase domain leading to activation of EGFR through trans-autophosphorylation of tyrosine residues in the carboxyl-terminal tail^23^. These phosphorylated residues recruit signaling molecules downstream of EGFR^23^. Our studies reveal that expression of the truncated form of EGFR is effective not only in impairing EGFR autophosphorylation induced by EGF but also receptor autophosphorylation induced by *T. gondii* infection. The lack of intracellular domains in DN EGFR would explain defective trans-autophosphorylation of full-length EGFR molecules that dimerize with truncated EGFR^12^. Src can also directly activate EGFR by causing Y845 phosphorylation of EGFR and recruitment of alternate signaling molecules^24,25^. Our studies revealed that the truncated form of EGFR also impairs EGFR Y845 phosphorylation induced by *T. gondii* infection. Given that *T. gondii* MIC3/6-mediated EGFR autophosphorylation and invasion-dependent Y845 EGFR phosphorylation occur almost simultaneously, it is possible that changes in conformation in the kinase domain induced by ligand (MIC)-mediated EGFR activation may facilitate the ability of Src to phosphorylate Y845.

*T. gondii* circulates in blood within infected leukocytes as well as extracellular tachyzoites and uses a hematogenous route to reach the CNS^3–5^. The fact that parasite load in the brain and retina were decreased in infected Trg-DN EGFR mice since the early stages of invasion of neural tissue, together with lower parasite load after i.v. injection of infected leukocytes or extracellular tachyzoites revealed that the protective effect of expression of DN EGFR is mediated by diminished invasion of the brain and retina. *T. gondii* has been proposed to invade the brain via transmigration of infected CD11b^+^ and CD11c^+^ cells (“Trojan horse” mechanism) or transmigration of extracellular tachyzoites (paracellular entry)^3,4,26^. However, there was no detectable difference in the transmigration of CD11b^+^ and CD11c^+^ cells into the brain parenchyma and the recruitment of leukocytes into the brain at a time (day 7 post-infection) when *T. gondii* load was already reduced in Trg-DN EGFR mice. While at day 14 post-infection Trg-DN EGFR mice have significantly lower inflammation in the CNS, it is unlikely that modulation of recruitment of infected leukocytes into the CNS plays a major role in EGFR-dependent regulation of invasion since the presence of *T. gondii* in blood of infected mice is short-lived and is no longer detected on day 10 post-infection^5^. *T. gondii* has also been proposed to invade the brain via transmigration of extracellular tachyzoites (paracellular entry)^3,4,26^. However, type II strains of *T. gondii* (like the ME49 and PTG strains used in these studies) have poor ability to transmigrate across host cell monolayers^26^. More recent work revealed that infected endothelial cells enable parasite release into the brain parenchyma (transcellular entry), and thus function as portal of entry into the CNS^5^. Indeed, our studies indicate that expression of DN EGFR in endothelial cells markedly reduced the foci of infected brain endothelial cells. While circulating extracellular tachyzoites are reported to infect brain endothelial cells^5^, our studies indicate that infected leukocytes also cause endothelial cell infection. In this regard, adhesion of infected leukocytes to endothelial cells leads to parasite egress that may lead to infection of endothelial cells^27^. Altogether, this work indicates that EGFR-dependent modulation of parasite survival within endothelial cells affects invasion of neural tissue.

Expression of DN EGFR in endothelial cells inhibited EGFR signaling, causing parasite targeting by LC3^+^ structures and killing of *T. gondii* dependent on the autophagy protein ULK1 and lysosomal enzymes. Moreover, *in vivo* expression of DN EGFR increased the percentages of parasite foci that were surrounded by LC3 and LAMP-1 in neural endothelial cells, and inhibition of autophagy prevented Trg-DN EGFR mice from reducing invasion of the CNS. These findings suggest that *in vivo* blockade of EGFR signaling in endothelial cells may decrease parasite invasion of the CNS by inducing anti-*T. gondii* activity in these cells. Noteworthy, autophagy proteins promote protection against cerebral and ocular toxoplasmosis^28^. Although less frequent than in Trg-DN EGFR mice, LC3 and LAMP-1 recruitment around *T. gondii* was noted in brain endothelial cells from Trg-Ctr mice, and 3-MA appeared to increase CNS invasion in these animals. These findings raise the possibility that some level of autophagic targeting of the parasite in endothelial cells may occur even when EGFR signaling is intact. Of potential relevance, CD40 ligation in endothelial cells induces autophagic killing of *T. gondii* even if these cells express WT EGFR^22^. Ongoing studies are examining the role of CD40 in parasite invasion of the CNS. Regardless of whether there are other mechanisms that regulate this process, the current work identified EGFR as a modulator neural tissue invasion by *T. gondii*. Of relevance to our studies, EGFR has been linked to the ability of *Escherichia coli* to gain access to the CNS^29^. However, the mechanism reported herein differs from that proposed for by *E. coli*. The bacteria triggers sphingosine 1-phosphate (S1P)-mediated activation of EGFR that in turn promotes bacterial invasion of brain endothelial cells^29^.

The CNS and peripheral organs such as the lung, spleen and liver are reported to exhibit different routes of invasion by *T. gondii*. Parasite detection in the lung and spleen precede detection in the blood following oral or i.p. routes of infection^16,30^. In this setting *T. gondii* appears to reach the lung and spleen through lymphatics rather than the bloodstream^30^. While infection of lung endothelial cells has been reported, those findings occurred after i.v. injection of free *T. gondii* tachyzoites. Moreover, Tie1 expression in lymphatics is restricted to their valves in adult mice^31^ suggesting that the reason why invasion of the peripheral organs is not affected in Trg-DN EGFR mice would be that most of the intraluminal surface of lymphatics would not express mutant EGFR.

In summary, our studies identified EGFR as an important modulator for the development of cerebral and ocular toxoplasmosis and uncovered EGFR as regulator of neural tissue invasion by *T. gondii*. These findings may be relevant to patients with ocular or cerebral toxoplasmosis since hematogenous spread of infection likely occurs in them^32,33^ and may explain why new ocular lesions can develop away from old retinal scars^34^. In addition, EGFR is expressed in various non-hematopoietic cells as well as microglia further supporting the possibility that administration of EGFR tyrosine kinase inhibitors may assist in the treatment of toxoplasmosis. Finally, the mechanism of protection reported herein may also be operative against other pathogens that invade the CNS, engage EGFR and appear to be controlled by autophagy such as *Neisseria* and *E. coli*^35^.

## Methods

All data generated or analyzed during this study are included in this published article (and its Supplementary Information files).

### Generation of Transgenic Mice and Ethics Statement

The responder line consisted of homozygous mice that express the tetracycline operator (*tetO*) upstream of a dominant negative (DN) mutant of EGFR that lacks the cytoplasmic tail (The Jackson Laboratory)^11^. The driver line consisted of heterozygous mice that express Tet-repressible transactivator (tTA) under the control of the *Tie1* promoter (provided by Nicole Ward, Case Western Reserve University)^9^. Transgenic mice were on a B6SJL background. Both lines of transgenic mice were bred while receiving doxycycline-containing food. Doxycycline was removed at birth. Offspring were identified using PCR analysis of genomic DNA using the following primers: TetO-DN EGFR Forward: 5′-ATCCACGCTGTTTTGACCTC-3′; Reverse: 5′-TGCCTTGGCAGACTTTCTTT-3′ (The Jackson Laboratory); Tie1-tTA Forward: 5′-CTCACTTTTGCCCTTTAGAA-3′, Reverse: 5′-GCTGTACGCGGACCCACTTT-3′. Littermates that inherited only one transgene (single transgenic and non-expressing) were used as controls for the double transgenic offspring. Mice were bred and kept at the Animal Resource Center (Case Western Reserve University). The recommendations from the Guide for the Care and Use of Laboratory Animals and the National Institute of Health were followed in this study. The protocol was approved by the Institutional Animal Care and Use Committee of Case Western Reserve University School of Medicine (Protocol Number 2015-0130).

### *In vivo* infection with *T. gondii*

Female mice (6 to 10 weeks of age) were infected i.p. with 30 cysts of the ME49 strain of *T. gondii*. In certain experiments, mice were infected i.v. with 5 × 10^5^ tachyzoites of the PTG strain of *T. gondii* or received i.v. 1 × 10^5^ dendritic cells that had been previously infected with PTG *T. gondii* tachyzoites. 3-methyl adenine (Sigma Chemical; 10 mg/kg) or vehicle was administered i.p. once daily beginning 1 d prior to challenge with *T. gondii*-infected cells. Mice were perfused prior to euthanasia.

### Mammalian cells and *in vitro T. gondii* infection

To isolate endothelial cells, lungs and brains were digested with collagenase A (1 mg/ml) and DNAse (100 μg/ml; Worthington Biochemicals) using gentleMACs C tubes and a gentle MACS Dissociator (Miltenyi Biotec). Homogenates were passed through a 40 μm cell strainer. Leukocytes were depleted using CD45 immunomagnetic beads (Miltenyi Biotec) followed by positive selection for endothelial cells using anti-CD31 or anti-CD146 MicroBeads (Miltenyi Biotec). Endothelial cells (>90% CD31^+^) were grown in medium containing Endothelial Growth Supplement (EGS; Sigma Chemical). Endothelial cells were challenged with *T. gondii* tachyzoites of the RH strain and the percentages of infection, the numbers of vacuoles and tachyzoites per 100 cells were determined using light microscopy by counting at least 200 cells per monolayer. The dendritic cell line DC2.4 (gift from Kenneth Rock, University of Massachusetts) was infected with PTG *T. gondii*. Extracellular tachyzoites were removed by extensive washing at 1 h and 18 h after challenge followed by i.v. administration into mice.

### Histology and immunohistochemistry

Four 5 μm sections from different areas of the brain and eye were stained by periodic acid Schiff hematoxylin (PASH) or hematoxylin and eosin stain respectively. Histopathologic changes (Brain: microglial nodules, perivascular and diffuse inflammation; Retina: disruption of architecture, perivascular and vitreal inflammation) were scored from 0 to 4 similar to previously described criteria^36,37^. Sections were also incubated with anti-*T. gondii* Ab (BioGenex) and Tomato lectin-DyLight 488 (Vector laboratories) that efficiently labels neural endothelial cells^38^ (at 0.5 μg/ml it stains neural endothelial cells rather than microglia) or anti-CD31 Ab (Elabscience). Coronal sections at the septo-diencephalic region (level of the thalamus) were examined at X400. The numbers of clusters of *T. gondii* parasites within tomato lectin^+^ elongated structures (endothelial cells) were counted per whole coronal section. Brain sections were also stained with anti-LC3 (Abgent) or anti-LAMP-1 (Developmental Studies Hybridoma Bank) Abs. Accumulation of LC3 or LAMP-1 around *T. gondii* located in endothelial cells was defined as the presence of a ring-like structure that surrounds the parasite^21,22^. Slides were analyzed using Leica DMI 6000 B automated microscope equipped for epifluorescence microscopy.

### Real-time quantitative PCR

Genomic DNA was isolated using the DNeasy kit (QIAGEN). *T. gondii B1* gene expression was measured by real-time quantitative PCR using SYBR green PCR Master Mix. *L32* was used as housekeeping gene. Parasite load/μg of genomic was calculated using a standard curve of DNA from 1 to 10^5^ tachyzoites of the ME49 strain per reaction. RNA isolated with the RNeasy kit (QIAGEN) was reverse transcribed (Quantitect Reverse Transcription kit; QIAGEN) and cDNA was used as template for RT-PCR using the SYBR Green PCR Master Mix and primers for IFN-γ, IL-12 p40, TNF-α, NOS2 and 18S rRNA^28^. Samples were run in triplicates using a StepOne Real Time PCR system (Applied Biosystems). Samples were normalized to the 18S rRNA housekeeping gene.

### Flow cytometry

BMNC isolated as described^28^ were stained with anti-CD3, anti-CD4, anti-CD8, anti-CD11b, anti-CD11c, anti-CD45, anti-F4/80, anti-Gr-1 or appropriate isotype control mAb (all from eBiosciences). CFSE (0.7 mg in 0.3 ml PBS-DMSO 7%; Santa Cruz Biotechnologies) or vehicle were injected i.v. on day 5 post-infection as reported^3^. BMNC were then collected and stained with anti-CD11b, anti-CD11c or isotype control mAb (eBiosciences) and examined for expression of CFSE. Splenocytes were incubated with or without anti-CD3 mAb and Brefeldin A (10 μg/ml; eBiosciences). Cells were stained with anti-CD3, anti-CD4 and anti-CD8 (eBiosciences), permeabilized, stained with anti-IFN-γ or isotype control mAb (eBiosciences) and analyzed in an LSR II flow cytometer (BD Biosciences).

### ELISA and nitric oxide assays

Concentrations of IL-12 p40, IFN-γ and TNF-α (R&D Systems) were measured in sera. Splenocytes (2 × 10^6^/ml) were incubated with *T. gondii* lysate antigens (TLA, 10 μg/ml) and the supernatants were collected at 24 h for detection of IL-12 p40 and TNF-α or at 72 h for IFN-γ and measurement of nitrite concentrations using the Griess reaction assay (Promega Corporation). Anti-*T. gondii* IgG was examined by ELISA. The titer of antibody was calculated by determining the highest dilution of serum that yielded a reading higher than the mean plus 2SD of the reading in the sera from uninfected mice.

### Transfections

Cells were transfected with ULK1 siRNA or control siRNA (Life Technologies) using Lipofectamine 2000 (Invitrogen, Carlsbad, CA).

### Immunoblot

PVDF membranes were probed with antibodies against Irgm3, actin (both from Santa Cruz Biotechnologies), extracellular domain of EGFR (R & D Systems), intracellular domain of EGFR (Santa Cruz Biotechnologies), phospho-Y845 EGFR (Cell Signaling), phospho-Y1068 EGFR (Invitrogen) and ULK1 (Sigma-Aldrich) followed by incubation with secondary antibodies conjugated to horseradish peroxidase (Santa Cruz Biotechnologies). All images were generated using Photoshop and, when necessary, they were subjected to processing that was limited to change in brightness and/or contrast. These changes were applied equally to the whole image.

### Immunofluorescence

Endothelial cells challenged with RFP *T. gondii* were incubated with LC3 antibody (MBL International), or anti-LAMP-1 (Developmental Studies Hybridoma Bank) followed by secondary antibodies. Accumulation of LC3 or LAMP-1 around *T. gondii* was examined. At least 50 cells per well (duplicate or triplicate wells per group per experiment) were counted manually.

### Statistics

Statistical significance of the data was determined by performing the 2-tailed Student’s *t* test and ANOVA. Histopathologic changes were assessed by using the Mann-Whitney *U* test. Statistical significance was considered when *p < *0.05.

## Electronic supplementary material

Supplementary file
