# Supplementary material for: Epidermal growth factor receptor promotes cerebral and retinal invasion by Toxoplasma gondii
Source: Sci Rep. 2019 Jan 24;9:669. doi: 10.1038/s41598-018-36724-2 (PMC6345933; doi:10.1038/s41598-018-36724-2)

Epidermal growth factor receptor promotes cerebral and retinal invasion by *Toxoplasma gondii*

Yalitza Lopez Corcino[1,2], Jose-Andres C. Portillo[2], Carlos S. Subauste[1,2]*

[1]Department of Pathology, Case Western Reserve University School of Medicine, Cleveland, Ohio, USA

[2]Division of Infectious Diseases and HIV Medicine, Department of Medicine, Case Western Reserve University School of Medicine

Supplementary Figure 1. Trg-DN EGFR mice express truncated EGFR in endothelial cells. (**a**) Binary tetracycline (Tet) repressible system. (**b**) PCR of genomic DNA to examine expression of transgenes in WT (B6SJL), Trg-Ctr (single transgenic) or Trg-DN EGFR (double transgenic) mice. Arrow represents DN EGFR. (**c**) Expression of EGFR in endothelial and non-endothelial lung cells. Endothelial ($CD31^+$) and non-endothelial cells ($CD31^-$) were isolated from lungs of Trg-Ctr and Trg-DN EGFR mice and subjected to immunoblot using Ab directed against the intracytoplasmic domain of EGFR (only detects WT EGFR), Ab directed against the extracellular domain of EGFR (enables detection of truncated mutant that lacks intracytoplasmic EGFR) or Ab against actin. (**d**) Expression of EGFR in brain endothelial cells and splenocytes from Trg-Ctr and Trg-DN EGFR mice. Lysates were probed as above. (**e**) Endothelial cells from brains of Trg-Ctr and Trg-DN EGFR were stimulated with EGF (1 ng/ml). Expression of phospho Y1068 EGFR and total EGFR was assessed by immunoblot in lysates obtained at 15 min. Results are representative of 3 independent experiments.

Supplementary Figure 2. Effect of DN EGFR on the induction of a type 1 cytokine response and expression of Irgm3 and nitric oxide. Trg-Ctr and Trg-DN EGFR mice were infected with tissue cysts of the ME49 strain of *T. gondii*. (**a**) Serum was obtained at 5 days and used to measure levels of IL-12 p40, IFN-γ and TNF-α. (**b**) Splenocytes obtained on day 7 were incubated with or without *T. gondii* lysate antigens (TLA) and supernatants were collected to measure IL-12 p40, IFN-γ and TNF-α. (**c**) Splenocytes were incubated with TLA and supernatants were collected to measure nitric oxide by Griess reaction. (**d**) Spleens and lungs collected at day 7 were lysed subjected to

immunoblot using Ab against Irgm3 or actin. (**e**) Splenocytes were cultured with anti-CD3 mAb and the percentages of $CD3^+CD4^+$ or $CD3^+CD8^+$ T cells that became IFN-γ^+ were determined by flow cytometry. Bars are mean $\pm$ SEM of 9 samples per group.

Supplementary Figure 1

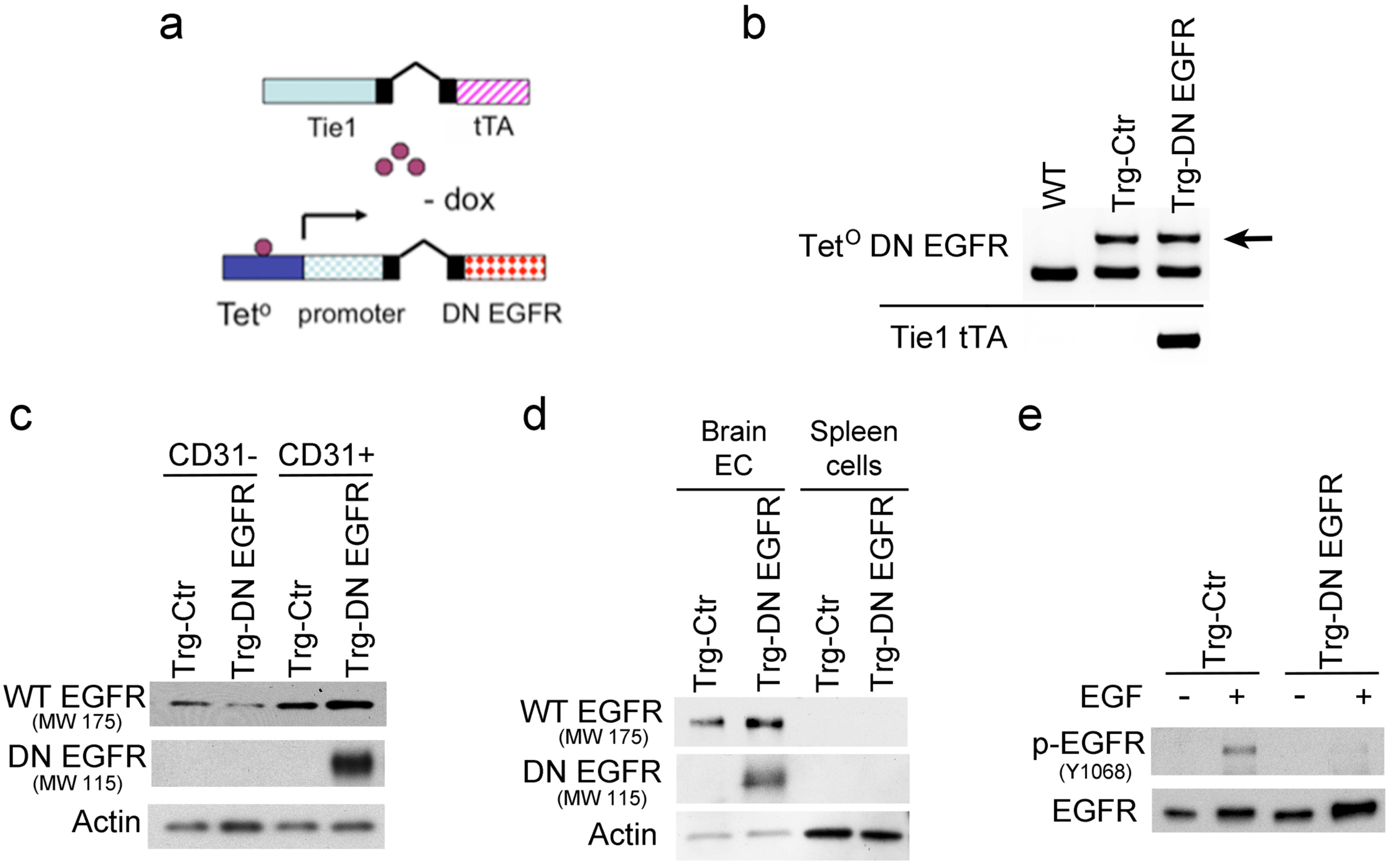

Supplementary Figure S2

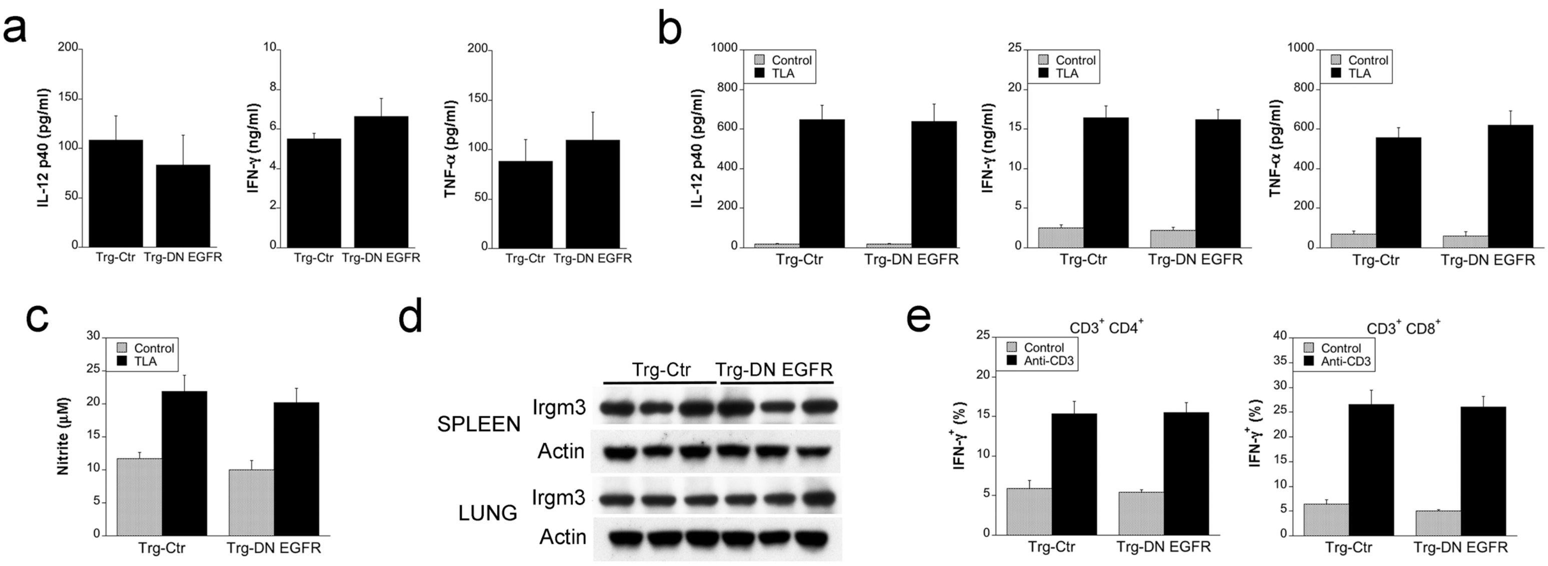

GELS FIGURE 6

6b

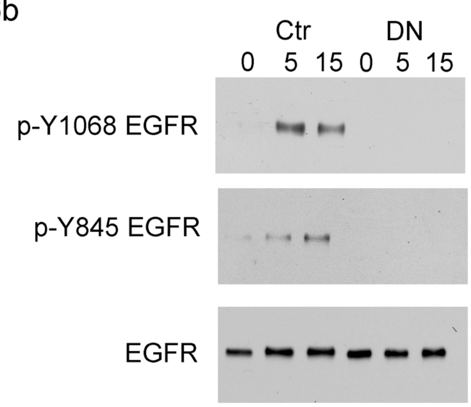

6f

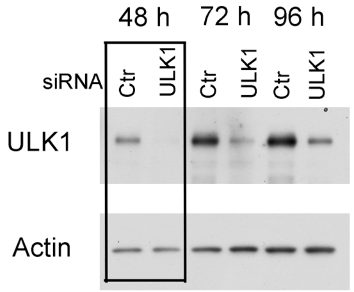

GELS FIGURE S1

S1b

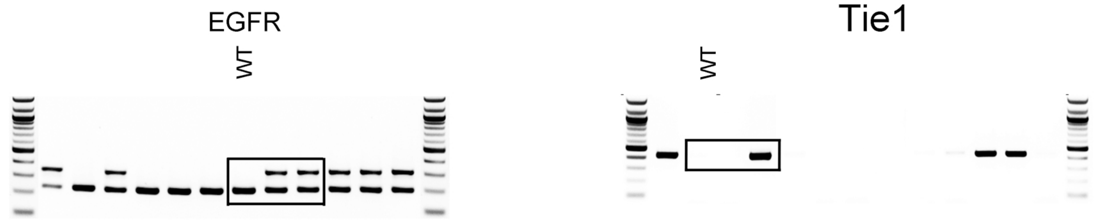

S1c

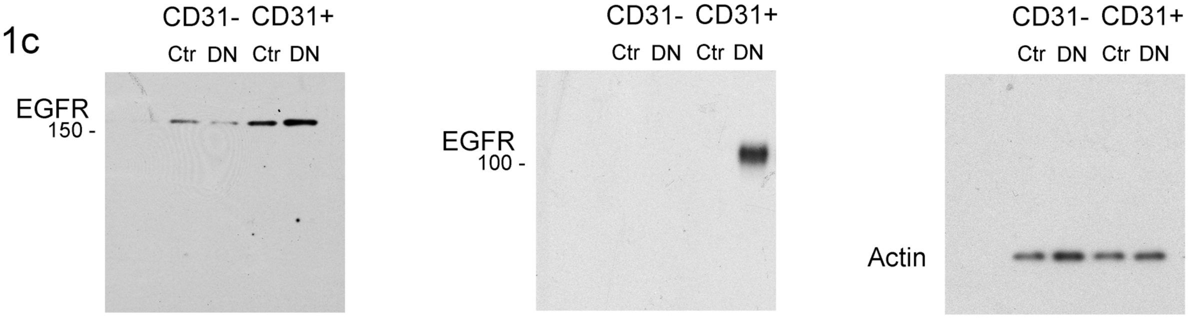

S1d

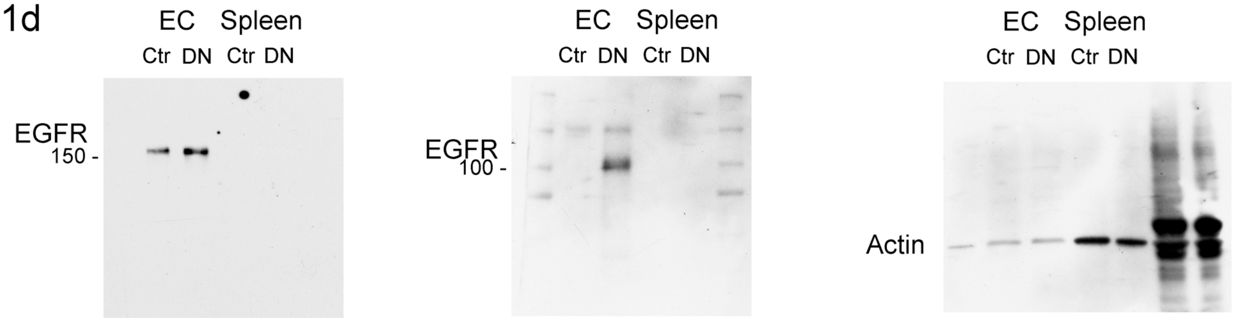

S1e

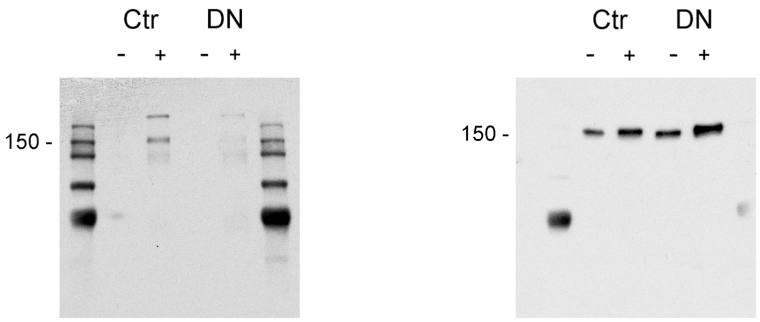

GELS FIGURE S2d

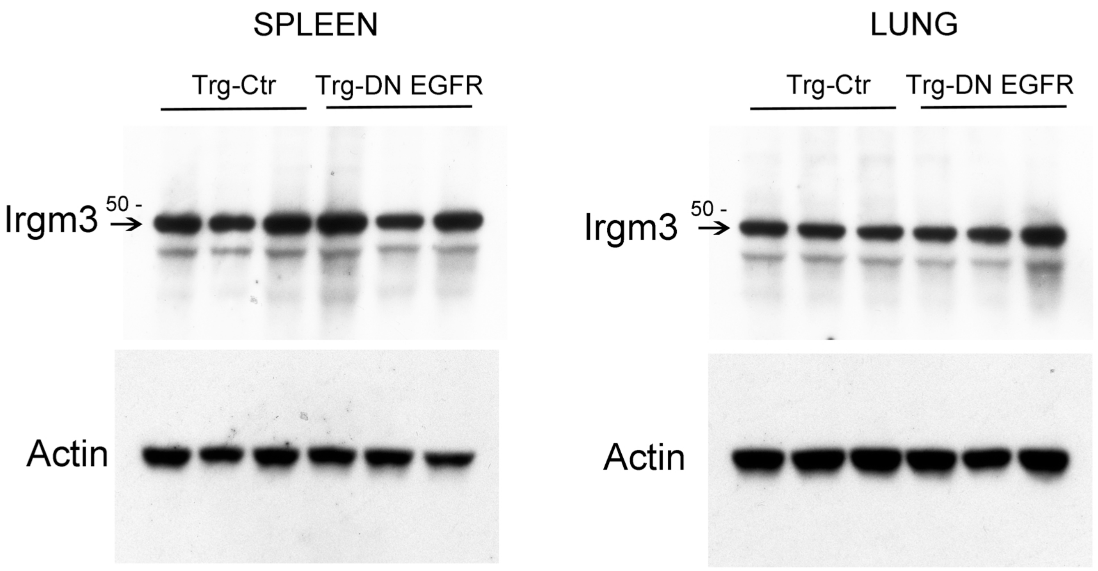

Supplement: Supplementary file 1 — Supplementary file [file 41598_2018_36724_MOESM1_ESM.pdf]
